# CD8^+^ T-Cell Mediated Control of HIV-1 in a Unique Cohort With Low Viral Loads

**DOI:** 10.3389/fmicb.2021.670016

**Published:** 2021-05-28

**Authors:** Amber D. Jones, Svetlana Khakhina, Tara Jaison, Erin Santos, Stephen Smith, Zachary A. Klase

**Affiliations:** ^1^Department of Biological Sciences, University of the Sciences, Philadelphia, PA, United States; ^2^The Smith Center for Infectious Diseases and Urban Health, West Orange, NJ, United States; ^3^Department of Pharmacology and Physiology, Drexel University College of Medicine, Philadelphia, PA, United States; ^4^Center for Neuroimmunology and CNS Therapeutics, Institute of Molecular Medicine and Infectious Diseases, Drexel University College of Medicine, Philadelphia, PA, United States

**Keywords:** HIV-1, HIV controller, Low Viral Load, CD8^+^ T cell, HLA compatibility, CD4:CD8 ratio

## Abstract

A unique population of HIV-1 infected individuals can control infection without antiretroviral therapy. These individuals fall into a myriad of categories based on the degree of control (low or undetectable viral load), the durability of control over time and the underlying mechanism (i.e., possession of protective HLA alleles or the absence of critical cell surface receptors). In this study, we examine a cohort of HIV-1 infected individuals with a documented history of sustained low viral loads in the absence of therapy. Through *in vitro* analyses of cells from these individuals, we have determined that infected individuals with naturally low viral loads are capable of controlling spreading infection *in vitro* in a CD8^+^ T-cell dependent manner. This control is lost when viral load is suppressed by antiretroviral therapy and correlates with a clinical CD4:CD8 ratio of <1. Our results support the conclusion that HIV-1 controllers with low, but detectable viral loads may be controlling the virus due to an effective CD8^+^ T-cell response. Understanding the mechanisms of control in these subjects may provide valuable understanding that could be applied to induce a functional cure in standard progressors.

## Introduction

Human immunodeficiency virus type-1 (HIV-1) infection causes disease by causing immunosuppression (Shearer, 1998; Elfaki, 2014). The degree of immunosuppression is closely and inversely linked to the concentration of CD4^+^ T-cells circulating in blood (Ingole et al., 2011; Merci et al., 2017). The average CD4^+^ T cell count is 1,000 cells/μL. HIV-1 infection causes a drop of 60 cells/uL per year on average (Schwartländer et al., 1993; Patrikar et al., 2014; Parsa et al., 2020). When the CD4^+^ T-cell count falls below 200 cells/uL, a person is at increased risk of opportunistic infections and malignancies (Egger et al., 2002; Institute of Medicine (US) Committee on Social Security HIV Disability Criteria, 2010; Opportunistic Infections Project Team of the Collaboration of Observational Hiv Epidemiological Research in Europe (Cohere) in EuroCoord, Young et al., 2012; Merci et al., 2017). Approximately 3–6 months after infection, plasma HIV-1 RNA concentration referred to as “viral load” (VL) reaches a steady state with a median of 40,000 copies/ml (c/ml). The level of viremia correlates to the rate of CD4^+^ T-cell loss (O’Brien W. A. et al., 1996; Mellors et al., 1997; de Wolf et al., 1997). CD4^+^ T-cells decrease ∼4% per year per log c/ml of HIV-1 RNA. The vast majority of people living with HIV (PLWH) have a VL > 1,000 c/ml of plasma. A very small percentage, < 1%, have a VL below the limit of detection, so-called undetectable.

Initially interested in studying the host factors of PLWH with naturally low viral loads (LVLs), we studied several individuals with VLs < 2,000 c/mL. As in published studies, isolated CD4^+^ T-cells from all LVLs supported HIV-1 *in vitro* growth. We next tested the ability of PHA-stimulated Peripheral Blood Mononuclear Cells (PBMCs) from LVLs to grow HIV-1. Surprisingly, the results segregated LVLs into two distinct groups, those with consistently undetectable VLs referred to as elite controllers (ECs) and those with VLs between 100–2,000 c/mL, which we refer to as LVLs. PHA-stimulated PBMCs from EC donors supported HIV-1 growth at a level similar to normal donor PBMCs. On the other hand, no growth could be detected in PBMCs from LVLs and we were able to associate this control with CD8^+^ T-cells.

CD8^+^ T-cells are known to play a critical role in the control of viral infection and their temporal appearance has been associated with a reduction in plasma viremia following acute HIV infection (Koup et al., 1994). To underscore the importance of CD8^+^ T-cells in the control of viral replication, previous studies have demonstrated that a robust rebound of plasma viremia results from antibody-mediated depletion of CD8^+^ T-cells from antiretroviral therapy (ART) treated simian immunodeficiency virus infected rhesus macaques (Cartwright et al., 2016; McBrien et al., 2020a,b). In addition, simian human immunodeficiency virus infected rhesus macaques who gained viral control when treated with combinatorial broadly neutralizing antibodies (bNAbs) became susceptible to infection when depleted of CD8^+^ T-cells, thus providing further evidence which supports the indispensable role of CD8^+^ T-cells in the control of viral replication (Nishimura et al., 2017). It was also observed that there was a minimal viral load which persisted in rhesus macaques who gained viral control when treated with bNAbs but their viral loads were so low they required detection via ultrasensitive assays (Nishimura et al., 2017). It was postulated that although negligible, low viral loads in these animals permitted a sustained CD8^+^ T-cell response sufficient to control infection which is not achieved when viral load levels are further suppressed by treatment with ART such that when ART is interrupted there is an inability of CD8^+^ T-cells to control viral replication (Nishimura et al., 2017). Therefore, in the presence of a low viral load, we speculate that CD8^+^ T-cells retain the ability to control viral replication.

In this study, we report on the clinical and cellular factors associated with LVL inhibition of HIV *in vitro* (IHI). IHI is only seen in donors who have a detectable, but low viral load and a “flipped ratio” or CD4:CD8 < 1. IHI is lost when viral load is suppressed by ART and is mediated by CD8^+^ T-cells in an HLA-restricted fashion. Our results support the conclusion that HIV-1 infected individuals with a low viral load set point controls the virus through a CD8^+^ T-cell response. This response fades quickly after viremia is suppressed, establishing that this mechanism is different than the mechanism(s) involved in the control of viremia in ECs. Understanding how these subjects are different from standard progressors with detectable CTL response may lead to the development of immunomodulatory therapies that can induce a functional cure.

## Results

### LVL Donor PBMCs Are Resistant to HIV Infection

**FIGURE 1:**
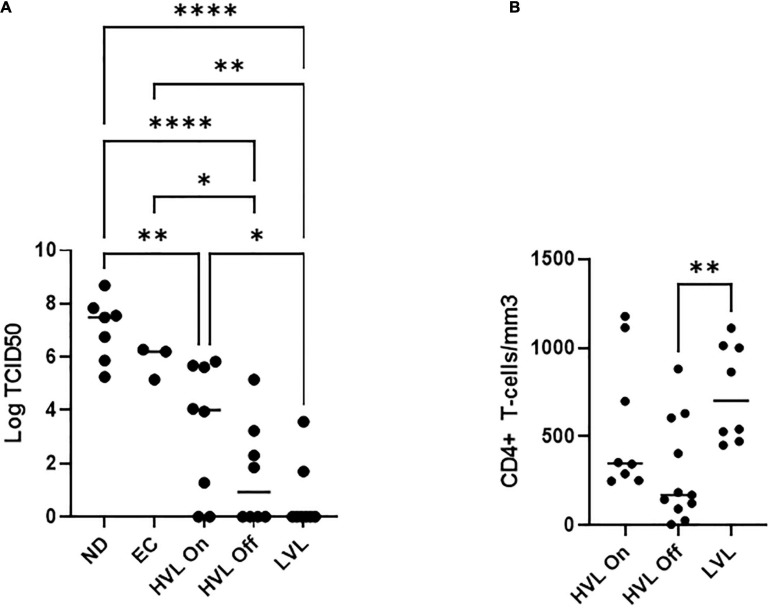
PBMCs from different classes of HIV-1 infected individuals show different susceptibility to *de novo* infection. **(A)** Limiting dilutions of NL4-3 were used to infect arrays of PBMCs from normal donors (ND), elite controllers (EC), high viral load on or off therapy (HVL On, HVL Off), or low viral load (LVL) donors in 96 well plates. p24 ELISA was used to determine productive infection in a given well and TCID50 was calculated. One-way ANOVA with Tukey’s multiple comparison was performed. *P*-value: ^∗^ < 0.05, ^∗∗^ < 0.01, ^****^ < 0.0001. **(B)** CD4^+^ T-cell counts in the blood of HVL (on/off therapy) and LVL. Mann-Whitney test was used to compare groups ^∗∗^*P* < 0.01.

**FIGURE 2:**
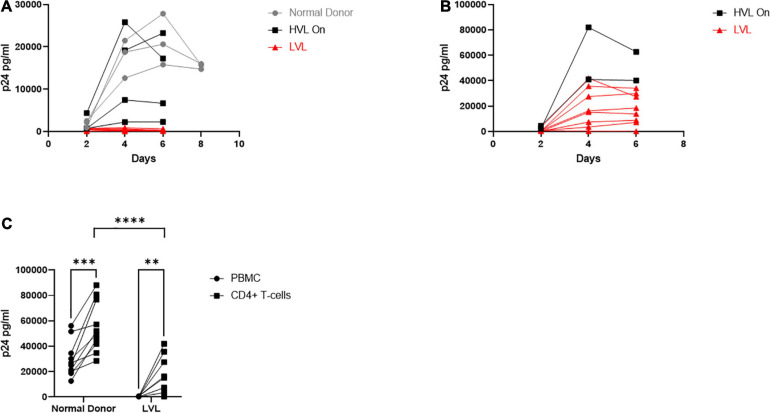
Total PBMCs from low viral load (LVL) donors do not support HIV spreading infection *in vitro*. **(A)** Total PBMCs collected from normal donors (gray, *n* = 3), high viral load donors on ART (HVL, black, *n* = 4) and ART naïve low viral load donors (LVL, red, *n* = 8) were induced with PHA and IL-2 then infected with NL4-3 48 h later. Supernatant was collected every 48 h post infection and virus levels were determined by p24 ELISA. **(B)** CD4^+^ T-cells were isolated from PBMCs from HVLs (black, *n* = 2) and LVLs (red, *n* = 8) by magnetic bead based isolation. Resulting CD4^+^ T-cells were induced with PHA and IL-2 then infected with NL4-3 48 h later. Supernatant was collected every 48 h post infection and virus levels were determined by p24 ELISA. **(C)** Measurement of peak p24 production in total PBMCs or isolated CD4^+^ T-cells from the indicated groups. Two-way ANOVA with Sidak’s multiple comparison test was performed. *P*-value: ^∗∗^ < 0.01, ^∗∗∗^ < 0.001, ^****^ < 0.0001.

For this study, we recruited ART naïve HIV-1 seropositive donors with viral loads below 2,000 copies/mL which we define as low viral load (LVL) donors, donors with a history of high viral loads (HVLs), elite controllers (ECs), or normal donors (Table 1). We performed a tissue culture infectious dose 50 (TCID50) assay to determine the relative susceptibility of PBMCs from each donor to infection using a common viral stock (Figure 1A). Normal donors and ECs showed the most susceptibility to infection *in vitro*. HVLs were less susceptible to infection, with no difference between HVLs suppressed on therapy and those that are therapy naïve. LVLs had the lowest average susceptibility to *in vitro* infection with HIV-1. However, LVLs were not statistically significant from HVLs off therapy. Blood CD4^+^ T-cell counts (Figure 1B) of LVLs remain high confirming that these donors control viral loads and maintain healthy CD4^+^ T-cell counts, unlike HVLs. We further analyzed the ability of PBMCs from normal donors, HVL donors on therapy and LVL donors to control spreading infection. PBMCs were activated with PHA and IL-2 for 48 h, washed, infected with NL4-3, and virus accumulation in the supernatant was measured every 2 days by p24 ELISA. Interestingly, PBMCs from LVLs suppressed *de novo* HIV-1 infection (Figure 2A). When the same assay was performed on uninfected normal donor and HVL donors PBMCs, we observed robust viral production by day four and six post-infection. As prior studies suggested, CD4^+^ T-cells from controllers (both LTNPs and ECs) could support viral replication (Cao et al., 1995; Sáez-Cirión et al., 2007; Julg et al., 2010; O’Connell et al., 2011), these data suggest that other cell types present in PBMCs may be mediating suppression in our system.

**TABLE 1:** Human subject classifications and clinical information.

**Classification**	**Patient ID**	**VL (copies/ml)**	**CD4 (cells/mm^3^)**	**CD8 (cells/mm^3^)**	**ART**	**Therapy**
HVL	ADV7140	<20	497	593	**ON**	Truvada/Tivicay
	BRW1143	<20	1,135	828	**ON**	Truvada/Tivicay
	CPU4801	<20	253	851	**ON**	Evotaz/Tivicay
	DER1295	<20	1,178	2,001	**ON**	Descovy/Tivicay
	ESN1170	<20	345	697	**ON**	Truvada/Tivicay
	HSA1084	<20	699	507	**ON**	Atripla
	QMY7270	<20	1,114	1,005	ON	Atripla
	WXM1008	<20	291	1,149	**ON**	Atripla
	XTD8730	30	251	764	**ON**	Tivicay/Reyataz/Norvir
	BPJ1299	9,600	605	1,202	Off	–
	CYJ1314	7,329	631	717	Off	–
	KLU1328	260,140	93	398	Off	–
	KMJ1960	30,000	145	1,367	Off	–
	UNJ7200	1,412,390	27	202	Off	–
	UTF9050	11,390	995	419	Off	–
	WBZ1300	24,000	170	1,206	Off	–
	ZDU1316	15,440	406	494	Off	–
LVL	FJG8070	250	1,070	1,434	Off	–
	FWU1270	100	688	ND	Off	–
	HCQ6670	80	488	506	Off	–
	MPY1313	1,550	473	756	Off	–
	NIM1164	750	723	987	Off	–
	SRS5930	230	699	792	Off	–
	VQY4910	750	723	987	Off	–
	AEM9650	1,630	1,001	2,081	Off	–
Normal donor	BTS1096	–	1,190	358	–	–
	CHT3368	–	360	646	–	–
	HFK1114	–	840	597	–	–
	YXC1164	–	1,061	757	–	–
	ND1	–	–	–	–	–
	ND2	–	–	–	–	–
	ND3	–	–	–	–	–
	ND4	–	–	–	–	–
Elite controller	EXT1011	<20	542	498	Off	–
	PQS6990	<20	1,148	827	Off	–
	RVF1231	<20	726	610	Off	–

### LVL CD4^+^ T-Cells Are Susceptible to the HIV Infection

The resistance of PBMCs from LVLs to infection could be due to many factors, including resistance to infection of CD4^+^ and/or CD8^+^ T-cell anti-HIV activity. To decipher between a CD4^+^ and CD8^+^ T-cell mediated phenotype, we performed an HIV-1 spreading infection assay on an isolated CD4^+^ T-cell population (Figure 2B). CD4^+^ T-cells were isolated from uninduced PBMCs from HVLs and LVLs using Miltenyi CD4^+^ T-cell negative isolation kit, purity of > 90% confirmed by flow cytometry and cells were activated and infected as above. All CD4^+^ T-cells isolated from LVLs were susceptible to HIV-1 infection except one (AEM9650) (Figure 2B). HLA typing revealed none of the HLA-B mutations commonly associated with viral resistance (data not shown). Comparing total virus output from infected PBMCs and CD4^+^ T-cells at days of peak replication, revealed that although CD4^+^ T-cells from LVLs supported HIV-1 infection, significantly less virus was produced compared to normal donors (Figure 2C).

### Control of Viral Replication *in vitro* by LVLs Is Mediated by CD8^+^ T-Cells

**FIGURE 3:**
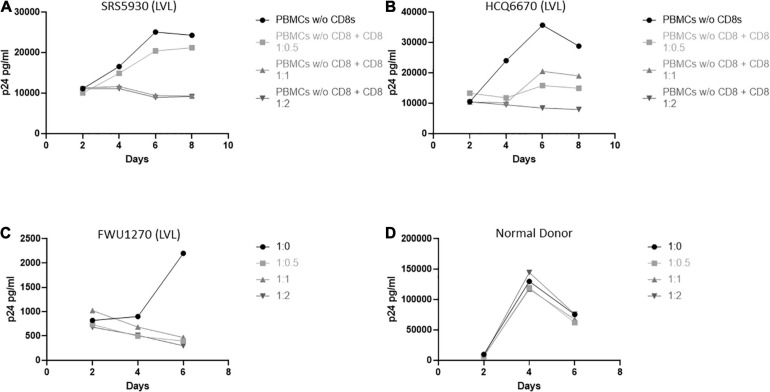
CD8^+^ T-cells are necessary and sufficient for control of viral replication by LVLs *in vitro.* **(A,B)** CD8^+^ T-cells were depleted from PBMCs of two LVLs. Depleted PBMCs and CD8^+^ T-cells were then stimulated for 48 h with PHA and IL-2 before being combined at the indicated ratio of PBMC:CD8 and infected with NL4-3. Supernatant was collected every 48 h post infection and virus levels were determined by p24 ELISA. CD4^+^ and CD8^+^ T-cells were isolated from PBMCs from **(C)** an LVL or **(D)** a normal donor. Isolated T-cells were induced with PHA and IL-2, combined as above (CD4:CD8) then infected with NL4-3 48 h later. Supernatant was collected every 48 h post infection and virus levels were determined by p24 ELISA.

We next investigated the role of CD8^+^ T-cells in the ability of LVLs to control viral replication *in vitro* (Figure 3). We depleted CD8^+^ T-cells from PBMCs from LVLs using a magnetic bead-based method. Depletion was verified by flow cytometry and we confirmed > 90% depletion of CD8^+^ T-cells. Cells were activated and PBMCs were infected as above. Depletion of CD8^+^ T-cells from PBMCs of two LVL donors resulted in susceptibility to HIV-1 infection (Figures 3A,B). Isolated CD8^+^ T-cells were also titrated back in to the PBMCs and this addback restored the suppressive phenotype at a ratio of 1:1 and 1:2 PBMCs:CD8s in one donor (Figure 3A) and at 1:2 PBMCs:CD8s in another donor (Figure 3B). We next tested whether CD8^+^ T-cells alone were sufficient for suppression of viral replication in CD4^+^ T-cells from an LVL. Populations of CD4^+^ and CD8^+^ T-cells were isolated from PBMCs of an LVL donor and a normal donor, activated and infected as above. As expected, isolated CD4^+^ T-cells were susceptible to viral replication (Figures 3C,D). Titration of CD8^+^ T-cells back with CD4^+^ T-cells at all ratios tested mediated suppression of viral replication from the LVL (Figure 3C) and not from the normal donor (Figure 3D). These results suggest that CD8^+^ T-cells are both necessary and sufficient to mediate the observed suppression of HIV-1 replication *in vitro*.

### HLA Compatibility Is Required for Induced CD8^+^ T-Cell Anti-HIV Function in Heterologous CD4^+^ T-Cells

**FIGURE 4:**
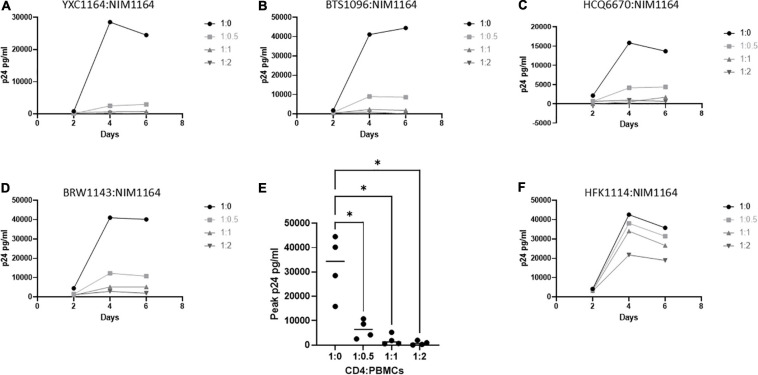
HLA compatibility is a requirement for LVL mediated HIV-1 suppression in heterologous CD4^+^ T-cells. PBMCs from LVL NIM1164 and isolated CD4^+^ T-cells from donors with varying degrees of HLA match were induced with PHA and IL-2. Heterologous CD4^+^ T-cell isolates from HLA-matched and mismatched donors and PBMCs from a LVL (NIM1164) were co-cultured at the indicated ratio of CD4:PBMCs and infected with NL4-3. Supernatant was collected every 48 h post infection and virus levels were determined by p24 ELISA. **(A)** YXC1164 is an HLA-matched normal donor primary relative of NIM1164. **(B)** BTS1096 is an HLA-matched normal donor. **(C)** HCQ6670 is a HLA-matched LVL. **(D)** BRW1143 is a HLA-matched HVL. **(E)** Measurement of peak p24 production from LVL HLA-matched donors at the indicated CD4:PBMC ratio. One-way ANOVA was performed. *P*-value: ^∗^ < 0.05. **(F)** HFK1114 is a HLA-mismatch normal donor.

It was previously reported by Killian et al., that an HLA class I match is required for HIV-1 inhibition by uninduced heterologous CD8^+^ T-cells (Mackewicz et al., 1998; Killian et al., 2018). In our donor cohort, we were able to identify several donors with matching HLA types (Table 2). To test the requirement of HLA compatibility for the ability of CD8^+^ T-cells from LVL donors to suppress HIV replication, we co-cultured activated PBMCs from an LVL donor with HLA-matched and non-HLA-matched isolated, activated heterologous CD4^+^ T-cells and infected as above (Figure 4). Of the LVLs in our study we chose to work with NIM1164 as this donor has matched HLA types with multiple other donors. We performed experiments to examine the ability of PBMCs from NIM1164 to suppress viral replication in isolated CD4^+^ T-cells from YCX1164, a primary relative containing several HLA matches (Figure 4A), BTS1096 a normal donor with matches in HLA-B, HLA-C, and DRB1 (Figure 4B), HCQ6670 a LVL with matches in HLA-A, HLA-B, and HLA-C (Figure 4C), BRW1143 an HVL with matches in HLA-B, HLA-C, and DRB1 (Figure 4D) and HFK1114 a normal donor with no matching HLA alleles (Figure 4F). The analysis of HIV-1 spreading infection in CD4^+^ T-cells and LVL PBMCs co-cultures at all CD4:PBMC ratios demonstrated marked inhibition of HIV-1 replication in cultures with matching HLAs compared to CD4^+^ T-cells alone (Figures 4A–E). Only minimal HIV-1 suppression was observed when LVL PBMCs were mixed with allogeneic CD4^+^ T-cells without matching HLA (Figure 4E). This suggests that HLA-class I compatibility is required for allogeneic suppression of HIV-1 replication by CD8^+^ T-cells from LVLs.

**TABLE 2:** HLA typing of subjects.

**Patient ID**	**Class**	**HLA-A**	**HLA-A**	**HLA-B**	**HLA-B**	**HLA-C**	**HLA-C**	**DRB1**	**DRB1**
NIM1164	LVL	24:02:00	30:01:00	14:02:01	42:01:01	8:02	17:01	1:02	3:02
HCQ6670	LVL	02:ANGA	30:01:00	42:01:01	57:03:01	17:01	18:02	08:AFPMU	13:AHUNK
BRW1143	HVL	32:01:00	33:03:00	42:01:00	44:03:00	4:01	17:01	3:02	13:ASWXB
BTS1096	ND	29:02:00	33:01:00	14:02	44:03:00	8:02	16:01	1:02	07:JDKZ
HFK1114	ND	30:02:00	34:02:00	15:03:01	44:03:01	2:10	4:01	4:05	15:03
YXC1164	ND	24:AVJRX	66:01:00	14:02	58:02:01	6:02	8:02	1:02	13:ASVAC

### Concurrent HIV Infection Is Required for CD8^+^ T-Cell Anti-HIV Function in LVL Donors

**FIGURE 5:**
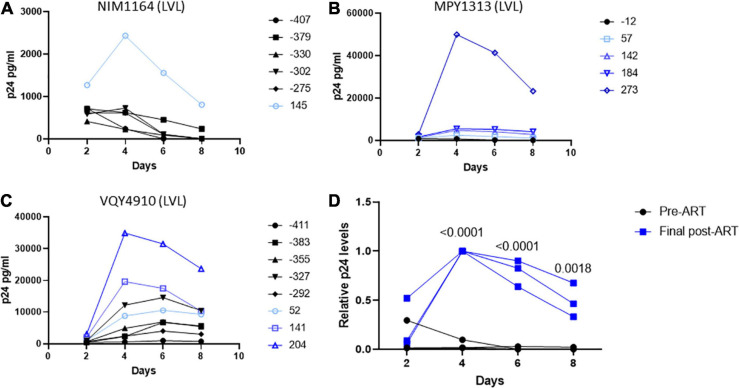
Suppression of viral load in LVLs by ART results in loss of *in vitro* control of viral replication. Longitudinal PBMC samples from LVLs **(A–C)** placed on ART. PBMCs were induced with PHA and IL-2 then infected with NL4-3 48 h later. Supernatant was collected every 48 h post infection and virus levels were determined by p24 ELISA. **(D)** Comparison of relative p24 levels pre- vs final post-ART for LVL donors. One-way ANOVA was performed. **(A–D)** Numbers indicate days post-ART. Black lines are pre-ART PBMC cultures and blue lines are post-ART.

During the duration of this study, several donors classified as LVLs were placed on ART (Table 3). To determine if the ability to control viral replication *in vitro* was related to viremia, we followed these individuals over time after initiation of therapy. PBMCs isolated from pre-therapy (black lines) and post-therapy (blue lines) were evaluated for their ability to support HIV-1 replication *in vitro* (Figure 5). For the LVL NIM1164, we observed suppression of viral replication in PBMCs for over a year prior to the start of therapy. ART successfully reduced viral load to undetectable levels (Table 3) and with this we observed low levels of viral replication *in vitro* (Figure 4A). A similar trend was observed with the LVL MPY1313 wherein therapy quickly suppressed viral load and a subsequent increase in the susceptibility of PBMCs to viral replication was observed. At 273 days post initiation of therapy, robust viral replication was measured *in vitro* (Figure 5B). These findings suggest that a detectable viral load is required for the observed control *in vitro.* In LVL VQY4910 a different pattern was observed. Following the start of ART an increase in the susceptibility of PBMCs to viral infection was observed over time (Figure 5C). For all three subjects PBMCs from the last time point sampled post-ART were incapable of supporting viral replication (Figure 5D). However, several pre-therapy samples for VQY4910 showed the ability to permit low level viral replication *in vitro*. This susceptibility fluctuated over time. Where data was available, we observed that time points that demonstrated viral control had a CD4:CD8 ratio in the blood of far <1 (Table 3). The samples with the greatest observed replication had a ratio close to one. Looking back at our other LVL samples that exhibited control, we also saw that these individuals had a CD4:CD8 ratio less than one, despite maintaining healthy CD4^+^ T-cell counts. This observation suggested that a subject must have both a detectable viral load and a CD4:CD8 ratio <1 in order to exhibit control *in vitro*.

**TABLE 3:** LVL subjects placed on ART.

**Patient ID**	**Draw**	**ART**	**Days on ART**	**Viral Load**	**CD4**	**CD8**	**CD4/CD8**
**NIM1164**							
	2	Off	–407				
	3	Off	–379	570	864	2,300	0.38
	4	Off	–330				
	5	Off	–302				
	6	Off	–275				
	7	On	145	<20	914	1,886	0.48
**MPY1313**							
	1	Off	–12	1,550	473	756	0.63
	2	On	57	<20	427	628	0.68
	3	On	142	<20	747	1,039	0.72
	4	On	184	<20	667	924	0.72
	5	On	273	<20	571	954	0.60
**VQY4910**							
	3	Off	–411	2,410	543	757	0.72
	4	Off	–383				
	5	Off	–355				
	6	Off	–327	2,410	528	570	0.93
	7	Off	–292				
	8	On	52	<20	895	710	1.26
	9	On	141	<20	728	554	1.31
	10	On	204	<20	1,025	735	1.39

### Pre-therapy CD8^+^ T-Cells From LVLs Are Capable of Suppressing Viral Replication in Post-therapy CD4^+^ T-Cells

**FIGURE 6:**
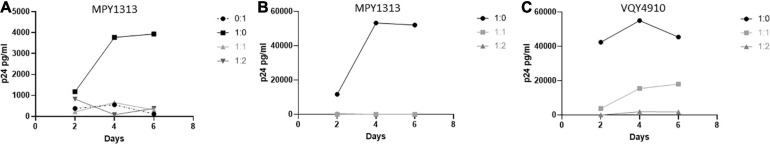
Pre-therapy CD8^+^ T-cells suppress replication in post-therapy CD4^+^ T-cells. Samples from before and after initiation of therapy were used to determine if pre-therapy samples could control viral replication in post-therapy samples. **(A)** PBMCs from MPY1313 and isolated CD4^+^ and CD8^+^ T-cells from **(B)** MPY1313 and **(C)** VQY4910 pre and post-therapy were mixed at the indicated ratios (post:pre) and infected with NL4-3. Supernatant was collected every 48 h and virus levels were determined by p24 ELISA.

We wanted to determine if CD8^+^ T-cells from pre-therapy, *in vitro* controlling, LVL donors could suppress viral replication in post-therapy samples that have lost the ability to control HIV-1 replication *in vitro*. To test this we activated and infected mixed PBMC cultures of MPY1313 from pre- and post-therapy time points with NL4-3 (post:pre; Figure 6A). As expected, post-therapy PBMC cultures alone supported and pre-therapy cultures alone suppressed viral replication. A mixture of post- and pre- therapy samples at 1:1 and 1:2 showed a complete suppression of viral replication. To confirm that this effect was specific to CD8^+^ T-cells as described above, we co-cultured activated, isolated CD4^+^ T-cells from post-therapy samples with activated, isolated CD8^+^ T-cells from pre-therapy samples and infected with NL4-3 (Donor MPY1313 and VQY4910; Figures 6B,C). CD8^+^ T-cells from pre-therapy samples successfully suppressed viral replication in CD4^+^ T-cells from post-therapy samples.

**FIGURE 7:**
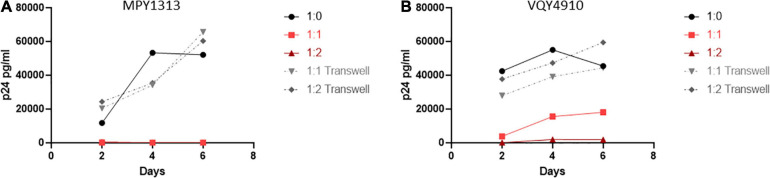
Cell contact is necessary to mediate viral suppression in LVLs. CD4^+^ T-cells were isolated from post-therapy samples from **(A)** MPY1313 and **(B)** VQY4910 and cultured with isolated CD8^+^ T-cells from the same donor pre-therapy. CD4^+^ and CD8^+^ T-cells were stimulated with PHA and IL-2 for 48 h before being combined in the given ratios (CD4:CD8) in direct contact (red solid lines) or in transwell (gray dashed lines). Cultures were infected with NL4-3, supernatant was collected every 48 h post infection and virus levels were determined by p24 ELISA.

To expand upon these findings and examine the need for cell to cell contact, we performed a similar experiment using a transwell setup to separate the isolated CD4^+^ and CD8^+^ T-cells (Figure 7). T-cell populations were isolated from MPY1313 and VQY4910 PBMCs. CD4^+^ T-cells from post-therapy time points were placed in a well and CD8^+^ T-cells from pre-therapy time points were either co-cultured directly with CD4^+^ T-cells or placed in transwell. Cultures were infected with NL4-3 and viral replication followed by measuring p24 levels in the supernatant over 6 days. Direct contact of CD8^+^ and CD4^+^ T-cells prevented viral replication. CD8^+^ T-cells in transwell were unable to mediate this effect, suggesting a requirement for cell to cell contact.

### Control of Infection Is Associated With Low Expression of the PD-1 Exhaustion Marker on CD8^+^ T-Cells

**FIGURE 8:**
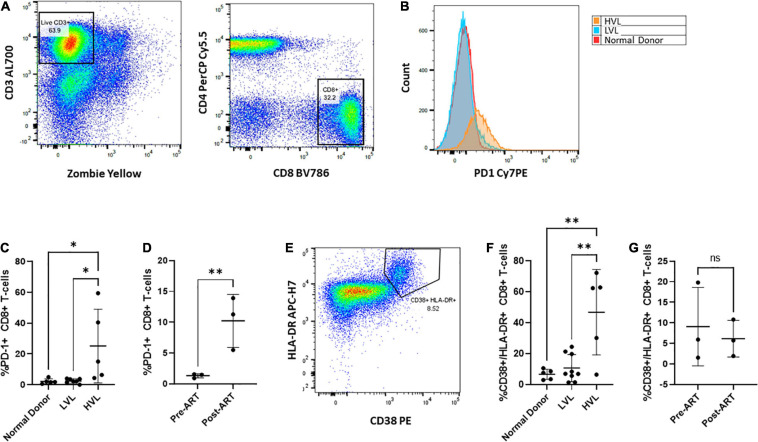
Phenotypic characterization of CD8^+^ T cells Pre- and Post-therapy. PBMCs from normal, LVL or HVL donors were stained for both T-cell exhaustion using PD-1 and immune activation using HLA-DR and CD38 and analyzed by flow cytometry. **(A)** Gating strategy for determining the exhausted population of CD8^+^ T-cells. Following gating to exclude debris and doublets, gates were drawn around live CD3 + T-cells and this population used to identify CD4-/ CD8^+^ T-cell populations. **(B)** Comparison between normal donor (red), ART naïve LVL donor (blue), and ART naïve HVL donor (orange) expression of PD-1. **(C)** Comparison of CD8^+^ T-cell expression of PD-1 between normal donors (*n* = 5), ART naïve LVLs (*n* = 7), and ART naïve HVLs (*n* = 5) statistical significance was determined by one-way ANOVA, ^∗^*P* < 0.05. **(D)** A longitudinal evaluation of CD8^+^ T-cell exhaustion pre- and post- therapy. A comparison of the expression of PD-1 was evaluated for 3 LVL donors both pre-ART and post-ART, ^∗∗^*P* < 0.01. **(E)** Immune activation was determined by gating CD8^+^ T-cells that expressed both HLA-DR and CD38. **(F)** Comparison of CD8^+^ T-cell expression of HLA-DR and CD38 between normal donors (*n* = 5), ART naïve LVLs (*n* = 7), and ART naïve HVLs (*n* = 5) statistical significance was determined by one-way ANOVA, ^∗∗^*P* < 0.01. **(G)** A longitudinal evaluation of CD8^+^ T-cell activation pre- and post-therapy.

We next sought to determine if control in LVLs was the result of measurable changes in CD8^+^ T-cells. Chronic infection has been associated with alteration of the activation of state of T-cells and the expression of markers of immune exhaustion (Wherry et al., 2007; Wherry and Kurachi, 2015; Saeidi et al., 2018). Increased expression of exhaustion markers such as PD-1 are associated with decreased CD8^+^ T-cell function (Day et al., 2006; Petrovas et al., 2006; Trautmann et al., 2006) and these markers have been seen in HIV infected individuals (Day et al., 2006; Petrovas et al., 2006; Trautmann et al., 2006; Sauce et al., 2011). We performed flow cytometry to assess the levels of PD-1 expressed on CD8^+^ T-cells from normal donors, HVL and LVL donors (Figure 8). In keeping with previously published studies, CD8^+^ T-cells from HIV + subjects with high viral loads had increased levels of PD-1. Our LVL subjects had PD-1 levels indistinguishable from normal donors (Figures 8B,C). To determine if expression of PD-1 correlates with control we examined the level of PD-1 expression on CD8^+^ T-cells from LVLs before the start of ART and the same subjects after ART when their PBMCs were able to support replication of HIV-1 in culture (Figure 8D). PD-1 levels were significantly higher in LVLs after the initiation of ART. We also performed flow cytometry to assess changes in immune activation by measuring the co-expression of HLA-DR and CD38 on CD8^+^ T cells from normal donors, LVLs, and HVLs (Figures 8E–G). Similar to what was observed with exhaustion, CD8^+^ T cells from HVLs with high viral loads had increased immune activation in comparison to both normal donors and LVLs (Figure 8F). To determine if immune activation also correlates with control, we evaluated the longitudinal co-expression of CD38/HLA-DR on CD8^+^ T cells both pre- and post-ART in our cohort of LVLs and determined that there was no significant difference in immune activation during times of *in vitro* control (pre-ART) in comparison to when PBMCs were susceptible to infection *in vitro* (post-ART) (Figure 8G).

## Discussion

Despite years of study we still do not completely understand what mediates natural control of HIV-1 infection in some individuals. Although studies have implicated multiple mechanisms that underlie many observed types of control, some mechanisms have not yet been elucidated. Our studies indicate that infected individuals with constantly low viral loads also demonstrate control of viral replication *in vitro* (Figure 2). This finding in itself provides another assay that could be performed to help further stratify types of control beyond available clinical observations.

Although absolute CD4^+^ T-cell count was initially used prognostically to assess progression to AIDS, it was observed that the rate of CD4^+^ T-cell decline varied significantly. Some individuals lost > 100 cells/μl per year, while others remained immunologically stable with normal CD4^+^ T-cell counts that sustained little to no loss over several years (Phair et al., 1992; Levy, 1993; Schwartländer et al., 1993; Buchbinder et al., 1994; Keet et al., 1994; Muñoz et al., 1995; Patrikar et al., 2014; Parsa et al., 2020). Before viral loads could be routinely measured, Dr. Ho et al. described a cohort of LTNPs who were HIV positive yet remained clinically asymptomatic without prolonged use of ART (Huang et al., 1995). These LTNPs, who were defined by CD4^+^ T-cell measurements and clinical outcomes, maintained normal CD4^+^ T-cell counts and had not developed opportunistic disease over an extended period of time. With the development of new technologies, focus shifted to quantifying plasma HIV-1 RNA as an independent reproducible marker of disease progression (Saksela et al., 1994; Mellors et al., 1995; Mellors et al., 1996). In 1997 Mellors et al., evaluated the rate of HIV disease progression in respect to both CD4^+^ T-cell count and plasma viremia. It was demonstrated that the rate of CD4^+^ T-cell loss was directly correlated to the level of HIV-1 viremia and together these parameters constituted the most accurate predictors of HIV-1 progression (O’Brien W. A. et al., 1996; Mellors et al., 1997; de Wolf et al., 1997). Those with high “viral loads” lost CD4^+^ T-cells more quickly than those with low or undetectable viral loads, who had little to no loss of CD4^+^ T-cells. Effective HIV-1 therapy confirmed that control of viremia prevented CD4^+^ T-cell loss and even allowed some CD4^+^ T-cell recovery in most cases. In support of this, it was found that the viral loads in LTNP cohorts varied from below average to undetectable (Pinto et al., 1995; García et al., 1997).

The measurement of viral load allowed further delineation of levels of control. Subjects who maintained low, but still detectable viral load remained LTNPs. Infected individuals with undetectable viral loads (<50 RNA copies/ml) began to be appreciated as a separate group (Clerici et al., 1992; Kelker et al., 1992; Cao et al., 1995; Pinto et al., 1995; Rowland-Jones et al., 1995; Fowke et al., 1996; Rowland-Jones et al., 1998; Lefrère et al., 1999; Pereyra et al., 2008). In the 2000’s, researchers began focusing on these subjects. This cohort, called elite controllers (EC) by some, has been studied by several groups (Shacklett, 2006; Dyer et al., 2008; Pereyra et al., 2008; Gonzalo-Gil et al., 2017; Pernas et al., 2018; Lopez-Galindez et al., 2019; Nguyen et al., 2019). Each showed that EC CD4^+^ T-cells supported HIV-1 *in vitro* growth as well as non-EC. Work on understanding the mechanism by which ECs suppress the virus has focused on CD8^+^ T-cells and the cytotoxic T-lymphocyte (CTL) response. Genetic analysis has found partially “protective” MHC alleles (Migueles et al., 2000). In these studies, isolated CD4^+^ T-cell were used exclusively (Rabi et al., 2011).

Previous studies, especially those examining ECs, have focused on the role of the CD8^+^ CTL response in controlling infection (Koup et al., 1994; Borrow et al., 1994; Barker et al., 1998; Wilkinson et al., 1999; Betts et al., 2001; Migueles and Connors, 2001; Sáez-Cirión et al., 2007; Betts et al., 2006; Yan et al., 2013; Cartwright et al., 2016; Nishimura et al., 2017; Nguyen et al., 2019; McBrien et al., 2020a,b). The PBMCs from our LVLs were resistant to infection, but isolated CD4^+^ T-cells were capable of supporting low level viral replication (Figure 2). Further experiments with isolated CD4^+^ and CD8^+^ T-cells revealed that CD8^+^ T-cells were both necessary and sufficient to mediate control of infection *in vitro* (Figure 3). The ability of LVL PBMCs to mediate suppression of viral replication in HLA-matched allogeneic CD4^+^ T-cells (Figure 4) and the requirement for cell to cell contact (Figure 7) strongly support that the observed control is due to classical CTL function. What remains to be determined is how LVLs maintain effective CD8^+^ T-cell functionality during HIV infection. That a detectable viral load remains in these individuals and the lack of known protective HLA alleles suggests this mechanism is different from what has been described for ECs (Migueles et al., 2000; Brener et al., 2015; Adland et al., 2018).

Unique to this study is the realization that the observed control *in vitro* is dependent upon the presence of detectable viral load in the donor and correlates with a low CD4:CD8 ratio (Figures 4, 5). This observation lead us to discover that HVL donors who had these characteristics (detectable plasma viral load and CD4:CD8 ratio < 1) also had PBMCs that were resistant to viral replication *in vitro* (Figures 5D,E). Our data does not indicate why this control in tissue culture correlates with successful clinical control in some donors but not others. The observation that LVLs can lose control over time (Figure 5) and that CD8^+^ T-cells from time points before the loss of control can suppress replication in post-control CD4^+^ T-cells (Figure 6) implies a change not in the target T-cell, but the functionality of the CD8^+^ T-cells.

The elucidation of the CD8^+^ T-cell driven mechanism of control in our LVL donors strongly supports the examination of viral replication *in vitro* as a way to further delineate types of clinical control. While it is possible that the lack of control in HVLs may be due to CTL escape of the virus, our examination of loss of control in LVLs suggests that the failure to control may be related to the subject’s immune system and not the virus. Our studies specifically highlight a role for changes in the level of CD8^+^ T-cell exhaustion and not immune activation. Although examination of changes over a greater period of time may reveal a role for T-cell activation. Extensive studies in the field have linked chronic immune activation to disease progress (Ascher and Sheppard, 1988; Fuchs et al., 1988; Fahey et al., 1990; Sheppard et al., 1991; Giorgi et al., 1993, 1999; Liu et al., 1997; Zajac et al., 1998; Hazenberg et al., 2003; Deeks et al., 2004; Hunt et al., 2008; Liovat et al., 2009) and more recent studies have examined T-cell exhaustion in chronic HIV infection (Day et al., 2006; Petrovas et al., 2006; Trautmann et al., 2006; Wherry et al., 2007; Sauce et al., 2011; Wherry and Kurachi, 2015; Saeidi et al., 2018). These two mechanisms go hand in hand and are strong candidates for explaining loss of control in LVLs over time as suggested by our evaluation of PD-1. Understanding this mechanism may lead to the development of interventions that could be used to induce a functional cure in people living with HIV.

## Materials and Methods

### Ethics Statement

The Smith Center for Urban Health and Infectious Disease, East Orange, NJ obtained written informed consent for the collection of blood donations from participating subjects. Samples were collected by trained medical staff under approved University of the Sciences’ protocol (IRB protocol 900702-3 and 797649-3).

### Human Subjects

All donors were recruited by the Smith Center for Urban Health and Infectious Disease. Two cohorts of HIV-1 infected donors were recruited, Low Viral Load (LVL) and High Viral Load (HVL) (characteristics at recruitment—Table 1). For the LVL cohort, 8 HIV-1 seropositive ART naïve donors were recruited, who were able to suppress HIV-1 infection independent of known protective HLA alleles. Four of these LVL donors were placed on combination ART during the course of this study. The HVL cohort included HIV-1 seropositive HVL donors pre- and post-therapy. Additionally, we recruited HIV-1 seronegative healthy control donors, Normal Donors that matched the age and characteristics of the two HIV-1 positive groups. For this study, classification as an HIV-1 controller required viremic control for a duration of at least 12 consecutive months in the absence of ART (O’Brien T. R. et al., 1996; Quinn et al., 2000; Gurdasani et al., 2014). HIV-1 controllers are defined as seropositive donors with viral loads of < 2,000 copies/mL and a CD4^+^ T-cell count > 500 cells/mm^3^ (O’Brien T. R. et al., 1996; Quinn et al., 2000; Gurdasani et al., 2014). HIV-1 seropositive donors that had low CD4^+^ T-cell counts, below 200 and had high viral load, above 5,000 copies/mL were designated as HVL donors.

### PBMC Isolation and Activation

PBMCs were purified from whole blood samples using Ficoll (GE Healthcare) gradient centrifugation and cryopreserved in 90% Fetal Bovine Serum (FBS; HyClone) containing 10% dimethyl sulfoxide (DMSO; Fisher). Frozen PBMCs were thawed and cultured in Roswell Park Memorial Institute (RPMI)-1640 complete media (GenClone) supplemented with 20% heat inactivated FBS, 1x penicillin-streptomycin-glutamine (PSG; Thermo Fisher Scientific), and 5% (5 U/mL) human rIL-2 (NIH AIDS Reagent Program). Cells were induced with 5 μg/mL phytohaemagglutinin-P (PHA-P) (Sigma) for 48 h at 37°C and 5% CO_2_.

### CD4^+^ and CD8^+^ T-Cell Isolation and Activation

CD4^+^ and CD8^+^ T-cells were purified from frozen PBMCs using MACS Miltenyi negative isolation kits (cat# 130-096-533 and cat# 130-096-495, respectively) according to manufacturer’s protocol. Enriched CD4^+^ and CD8^+^ T-cell populations were independently activated in RPMI complete media with 5μg/mL PHA for 48 h at 37°C and 5% CO_2_.

### HIV-1 Virus Stock

The HIV-1 stock used in this study was generated by transfecting pNL4-3 (NIH AIDS Reagent Program, ARP-2852, contributed by Dr. M. Martin) into HEK293T cells [American Type Culture Collection (ATCC, CRL-11268)] using TransFectin Lipid Reagent (BioRad, Cat# 1703351) following manufacturer’s instructions. Transfected cells were cultured in Dulbecco’s Modified Eagle’s Medium (DMEM) supplemented with 10% FBS and 1x PSG for 48 h at 37°C and 5% CO_2_. Virus containing supernatants were aspirated from the cells, filtered, and frozen in 1 ml aliquots. Frozen stocks were quantified by p24 Gag ELISA.

### Spreading Infection Assay

Activated total PBMCs, isolated CD4^+^ T-cells, or mixed CD4^+^ and CD8^+^ T-cells were infected with 17 ng/ml NL4-3 virus at 37°C and 5% CO_2_. For experiments where isolates were collected and recombined, cells were co-cultured for 3–6 h prior to infection. After 24 h, the virus containing media was removed and replaced. Virus production was evaluated by measuring the p24 levels in the supernatant using ELISA (Zeptometrix). Time points were collected every 48 h post infection for six to 8 days.

### Flow Cytometry

For phenotypic analysis of cell populations, all cells were washed with Phosphate Buffered Saline (PBS) without Ca^2+^ and Mg^2+^ (GenClone) and stained using fluorescently conjugated antibodies against the following cell surface markers following manufacturer’s instructions: CD3 (BD Biosciences (BD), Alexa700, clone SP34-2), CD4 (BD, PerCP-Cy5.5), CD8 (BD, BV786, clone RPA-T8) and CD279 (PD-1) (Invitrogen, clone eBioJ105), HLA-DR (BD, APC-H7, clone L243), and CD38 (BD, PE, clone HIT2). Cells were also stained for viability using the live/dead stain Zombie Yellow (BioLegend). Cell enumeration was carried out using Cytek FACSort DxP12 flow cytometer and data analysis using FlowJo v10.6.1 software.

## Data Availability Statement

The original contributions presented in the study are included in the article/supplementary material, further inquiries can be directed to the corresponding author/s.

## Ethics Statement

The Smith Center for Urban Health and Infectious Disease, East Orange, NJ obtained written informed consent for the collection of blood donations from participating subjects. Samples were collected by trained medical staff under approved University of the Sciences’ protocol (IRB protocol 900702-3 and 797649-3).

## Author Contributions

ZK and SS conceptualized this project. SK and AJ designed research, performed experiments, and analyzed and interpreted data. TJ assisted in experiments. AJ, SK, SS, and ZK wrote the manuscript. ZK aided in the design of the experiments and the analysis and interpretation of data. ES recruited patients, handled patient education and organization of clinical data. All authors contributed to the article and approved the submitted version.

## Conflict of Interest

The authors declare that the research was conducted in the absence of any commercial or financial relationships that could be construed as a potential conflict of interest.
